# Behavioural traits of individual homing pigeons, *Columba livia* f. *domestica*, in their homing flights

**DOI:** 10.1371/journal.pone.0201291

**Published:** 2018-09-27

**Authors:** Ingo Schiffner, Patrick Fuhrmann, Juliane Reimann, Roswitha Wiltschko

**Affiliations:** 1 Fachbereich Biowissenschaften, J. W. Goethe-Universität Frankfurt, Biologicum, Max-von-Laue-Straße 13, Frankfurt am Main, Germany; 2 Department of Ecology, Evolution and Behavior, The Hebrew University of Jerusalem, Givat Ram, Jerusalem, Israel; Bowling Green State University, UNITED STATES

## Abstract

Homing tracks of two groups of pigeons, *Columba livia* f. *domestica*, were analyzed in view of difference between individual birds and correlations between characteristic variables, looking at the initial phase while the pigeons were still at the release site, and the homing phase separately. Individual birds differed significantly in their flying speed during the initial phase, and one pigeon tended to stay longer at the release site than the others. There were no significant differences in steadiness and efficiency, indicating that all pigeons homed equally well. Differences in correlation dimension, a variable reflecting the complexity of the navigational process, reflect differences in the use of navigational information, with one bird apparently using less complex information than others. The flying speed during the initial phase was positively correlated with the flying speed during the homing phase. During the homing phase, the steadiness of flight and the efficiency of homing were closely correlated, and both tended to be positively correlated with the correlation dimension, suggesting that birds that use more complex navigational information home more efficiently.

## Introduction

Pigeons, *Columba livia* f. *domestica*, are social birds and normally fly in flocks. Yet since antiquity, they were used to carry messages, and were mostly released singly, documenting that each individual bird in principle has the ability to find its way home alone. Scientist began to approach this mysterious ability experimentally at the end of the 19^th^ century/beginning of the 20^th^ century (e.g. [1,2]). Initially, only two variables could be measured to assess the pigeons’ performance, namely *homing success*, given as the percentage of pigeons that returned, and *homing times*, the time each bird needed to return to the loft. In the 1950s, Matthews [3] and Kramer and v. St. Paul [4] discovered that displaced pigeons normally leave the release site in an oriented manner, that is, the birds agree about the direction in which they have to depart, mostly heading in the home direction or not far from it. This discovery allowed new approaches in pigeon research, since it introduced *vanishing bearings* and *vanishing intervals* as novel variables into orientation studies. In particular the bearings, usually measured with a compass when the pigeon vanished from sight of a good pair of binoculars, proved very useful, as they appeared to reflect the direction that the birds at a specific site considered to be their home direction. It made the assessment of the effect of various experimental manipulations possible, like e.g. shifting the birds’ internal clock or equipping them with magnets.

From the beginning of this century onward, the development of modern tracking devices, mostly based on GPS (e.g. [5–7]), provided even more information because they allowed the recording of the entire homing tracks with great precision. These recordings do not only document the position of a pigeon at any given time, but also its flying speed and its current heading. Mathematical analyses of these tracks enabled us to determine values like the *Point of Decision*, that is, the moment when the bird begins to leave the release site [8], and the *correlation dimension* which reflects the number of factors involved in the navigational process and thus allows an estimate of its complexity [9]. These new detailed data on the homing flights of pigeons also open up the possibility to look for differences in the homing behavior of individual birds.—In the present study, we use a novel approach to analyze the tracks of pigeons in view of possible individual traits, and their interrelations.

### Data to be analyzed

**Fig 1:**
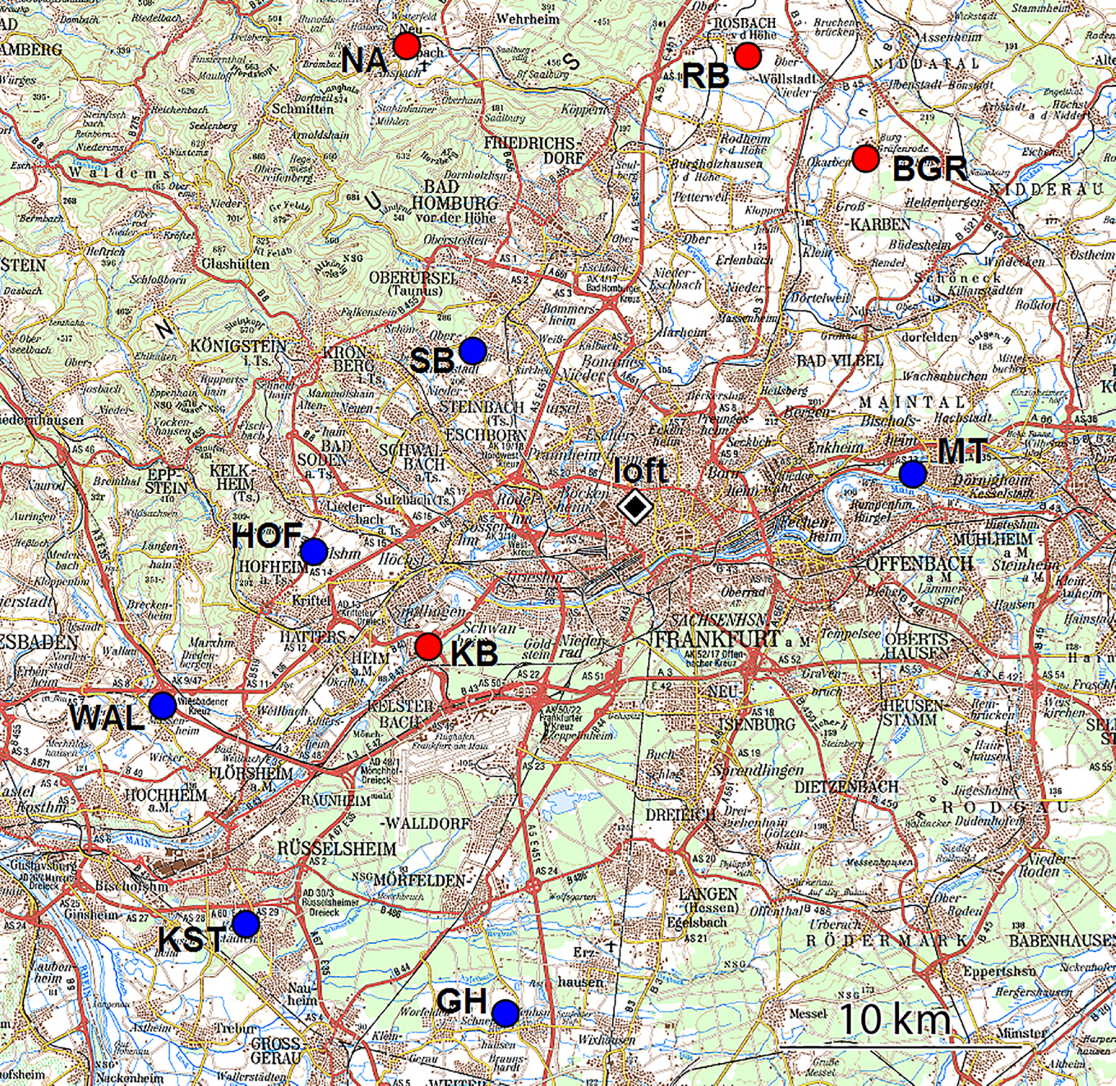
The release sites used. 2009 (blue) and 2010 (red). The home loft at Frankfurt am Main is marked by a black symbol near the center (for details, see S1 Table in Supporting Information) (underlying map: Top 50 Hessen).

When analyzing tracks in view of individual traits, a problem arises, because the tracks are also affected by external factors like e.g. the route taken, weather variables, temporal variations in the geomagnetic field and other environmental factors. For example, although we released the pigeons only on days with little or no wind, a certain effect of wind, in particular wind direction, on the flying speed cannot be excluded. The terrain crossed during homing and the amount of fluctuations of the magnetic field were also found to affect the tracks (see [10,11]). As a consequence, when looking for individual difference between birds, we can only compare tracks of birds that flew home in the same conditions, that is, from the same site on the same day, which limits the samples that can be analyzed. In our case it applies only to two groups of pigeons: 8 birds that homed from 6 sites in 2009 and 9 birds that homed from 4 sites in 2010. Their tracks form the basis of the present analysis; they are available on Movebank (movebank.org) and are published in the Movebank Data Repository [12]. The release sites lay between 9.3 and 23.5 km from the loft (Fig 1; for details, Supporting Information S1 Table).

The homing flights of pigeons can be divided into two phases, an *initial phase* and a *homing phase*. When pigeons are released, they usually do not take off right away, but linger around near the release site until, after a certain amount of time they reach a *Point of Decision* and begin their homing flight. This Point of Decision is characterized by an increase in flying speed and steadiness of flight; mostly, it is also associated with a change in direction, with the birds now heading closer to their home direction ([8]; for more details, Supporting Information S1 Text). The transition between *initial phase* and *homing phase* varies greatly between birds at the same site; it normally occurs 90 to 200 s after release and at distances of about 200–1000 m from the point of release (see e.g. [8,13]). If there were more than one Point of Decision, only the first one is considered in the present study to divide the track into two phases—the initial phase until the 1st Point of Decision and the homing phase thereafter–which are analyzed separately.

For the *initial phase*, we focus on the following variables:

1. *Duration* until the 1^st^ *Point of Decision* in seconds; it is roughly proportional to the distance flown during that time.
2. *Flying speed* in km/h until the 1^st^ *Point of Decision*, represented by its mean.
3. *Steadiness* of the flight during the initial phase, defined as the vector lengths resulting from all consecutive heading directions between the GPS fixes, with a vector length of 1 indicating a straight flight, whereas a vector length close to 0 means a highly tortuous flight, with little net distance covered.

The *homing phase* ends when a bird had approached the loft by 100 m. Here, we analyze the following variables:

4 *Flying speed* during the homing phase, as above.
5 *Steadiness* of the flight during the homing flight, defined as above.
5 *Efficiency* of the flight, defined as beeline distance release site—loft divided by the actual length of the route flown.
6 *Correlation dimension*, calculated by means of time lag embedding (see [9,10]), a method that is based on dynamic system theory [14] and the original algorithm proposed by Grassberger and Procaccia [15]. It indicates the number of degrees of freedom of a system and thus reflects its complexity, in case of the tracks of pigeons: the number of navigational factors involved, with a correlation dimension near 3 and above indicating true navigation (for details see Supporting Information S2 Text).

## Results and discussion

**Fig 2:**
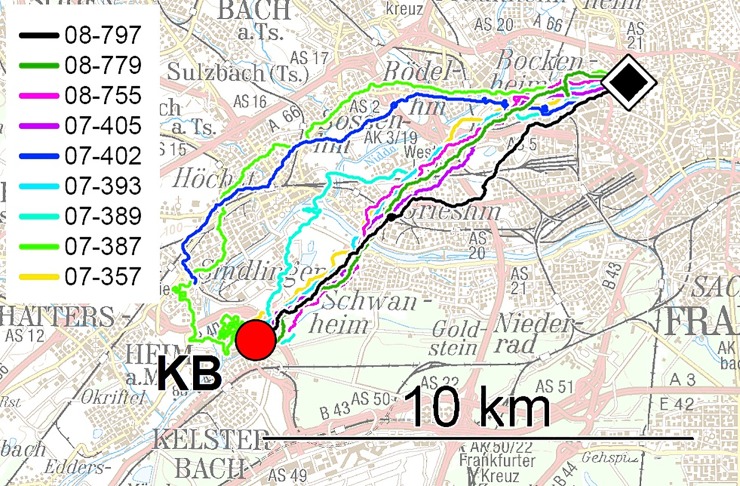
Example of tracks from the site KB. The release site,10.3 km from the loft, is marked by a red circle, the loft is indicated by a black symbol. The figure shows the tracks during the homing phase; those during the initial phase are not included because they overlap manifold. The various colors mark the tracks of different pigeons, see legend (underlying map: Top 50 Hessen:).

Fig 2 gives, as an example, the tracks of the 2010 birds released at the site KB; for the other tracks, see Supporting Information S1 and S2 Figs.

### Individual behavioral traits among pigeons

The medians of the variables analyzed for each individual pigeon and their range are listed in Table 1 for the initial phase and in Table 2 for the homing phas*e* (for the individual data, see Supporting Information S2 Table). They vary greatly: while the inter-individual variance between birds is considerable, the intra-individual variance, as documented by the range of the data, is even greater. There is no general difference between male and female pigeons (p > 0.05, Mann Whitney test for all variables)—both sexes master the task of homing equally well.

**Table 1:** Initial phase: Medians and, in parentheses, range of the data for individual pigeons.

Pigeon	Sex	Duration (s)	Speed (km/h)	Steadiness
Data of 2009, 6 flights per bird				
06–213	m	383 (45; 615)	56 (53; 59)	0.17 (0.07; 0.49)
06–214	m	143 (60; 405)	50 (43; 52)	0.22 (0.14; 0.46)
06–232	f	240 (45; 540)	51 (43; 52)	0.22 (0.09; 0.40)
06–233	f	180 (90; 390)	57 (51; 62)	0.22 (0.11; 0.42)
06–235	m	293 (120;1410)	60 (56;61)	0.26 (0.10;0.36)
06–243	m	113 (60; 180)	56 (52; 60)	0.36 (0.27; 0.67)
06–249	m	90 (45; 255)	49 (48; 56)	0.18 (0.12; 0.61)
06–254	f	150 (45; 870)	51 (48; 53)	0.28 (0.10; 0.43)
Medians	165	54	0.22	
Data of 2010, 4 flights per bird				
07–357	m	263 (180; 825)	53 (51; 54)	0.16 (0.08; 0.24)
07–387	f	218 (105; 345)	54 (47; 62)	0.24 (0.09; 0.38)
07–389	f	465 (210; 735)	55 (52; 61)	0.08 (0.07; 0.16)
07–393	f	368 (150; 405)	45 (43; 51)	013. (0.08; 0.48)
07–402	m	533 (240;1515)	58 (54; 61)	0.19 (0.06; 0.27)
07–405	m	210 (45; 405)	50 (45; 54)	0.18 (0.12; 0.65)
08–755	m	345 (75; 630)	60 (53; 64)	0.37 (0.04; 0.50)
08–779	m	75 (60; 150)	50 (48; 54)	0.22 (0.08; 0.24)
08–797	m	105 (45; 315)	56 (50; 60)	0.26 (0.12; 0.28)
Medians	263	54	0.19	

The 1^st^ column gives the pigeon’s individual number, with the first two digits indicating the year when it was born. *m*, *f*: male and female pigeons. The following columns give the median and, in parentheses, the minimum and the maximum recorded. *Duration* indicates the duration of the initial phase in seconds from the moment of release to the first Point of Decision; *Speed* gives the average flying speed during the initial phase in km/h; *steadiness* indicates the steadiness of the flight, given as mean vector lengths resulting from the headings of the various 1s-parts between two fixes.

**Table 2:** Homing phase: Medians and, in parentheses, range of the data for individual pigeons.

Pigeon		Speed (km/h)	Steadiness	Efficiency	Correl. dimension
Data of 2009, 6 flight per bird					
06–213	m	59 (55; 68)	0.77 (0.51; 0.93)	0.80 (0.47; 0.92)	3.80 (2.78; 4.30)
06–214	m	57 (48; 69)	0.75 (0.64; 0.88)	0.76 (0.60; 0.89)	3.27 (2.93; 5.03)
06–232	f	57 (52; 64)	0.69 (0.40; 0.88)	0.67 (0.46;0.88)	3.13 (2.68; 4.92)
06–233	f	61 (55; 64)	0.79 (0.44; 0.90)	0.79 (0.46; 0.90)	3.40 (2.47; 4.03)
06–235	m	63 (54; 65)	0.61 (0.48; 0.90)	0.63 (0.47; 0.90)	2.67 (2.44; 3.32)
06–243	m	57 (52; 70)	0.81 (0.36; 0.90)	0.80 (0.41; 0.90)	3.32 (3.10; 3.71)
06–249	m	58 (54; 63)	0.69 (0.48; 0.94)	0.66 (0.52; 0.94)	3.02 (2.60; 3.60)
06–254	f	55 (53; 64)	0.86 (0.65; 0.88)	0.86 (0.69; 0.88)	3.62 (3.31; 4.05)
Medians		58	0.76	0.78	3.30
Data of 2010, 4 flights per bird					
07–357	m	64 (62; 64)	0.84 (046; 0.86)	0.85 (0.48; 0.86)	3.48 (2.31; 3.60)
07–387	f	65 (63; 70)	0.58 (0.39; 0.72)	0.59 (0.40; 0.74)	3.31 (2.93; 3.56)
07–389	f	67 (62; 69)	0.73 (0.62; 0.90)	0.75 (0.63; 0.91)	3.37 (2.78; 3.86)
07–393	f	64 (55; 70)	0.99 (0.50; 0.94)	0.89 (0.52; 0.94)	3.86 (3.18; 5.22)
07–402	m	68 (67: 68)	0.75 (0.70; 0.79)	0.76 (0.72; 0.80)	3.16 (2.83; 4.18)
07–405	m	63 (61; 69)	0.60 (0.49; 0.84)	0.64 (0.56; 0.85)	3.57 (2.77; 3.75)
08–755	m	69 (65; 72)	0.72 (0.61; 0.85)	0.74 (0.61; 0.86)	3.25 (3.10; 3.44)
08–779	m	67 (64; 69)	0.83 (0.74; 0.89)	0.85 (0.74; 0.90)	3.62 (3.50; 4.25)
08–797	m	70 (68; 71)	0.74 (0.61; 0.91)	0.76 (0.61; 0.92)	3.21 (2.87; 3.47)
Medians		67	0.74	0.76	3.37

As in Table 1. *Efficiency* indicates the efficiency of th*e* homing flight, defined as direct distance to home divided by the actual length of the track; *Correl*. *dimension* indicates the correlation dimension of the homing flight (see Schiffner et al. 2011a).

The ART ANOVA indicates some significant differences between birds in some of the variables. They are given in Table 3 in bold print and concern the flying speed during the initial phase, where differences are observed in both groups. For the duration of the initial phase, difference are only indicated among the 2010 birds, and for speed during the homing phase and the correlation dimension, differences are only indicated among the 2009 birds. -Table 4 lists the difference between individual birds by least squared means test using the Tukey method for multiple comparisons, with the left column starting with the highest values, the right column with the lowest.

**Table 3:** Differences between individual birds: Results of the ART ANOVA.

Variable	F_7,35_	sign?	F_8,24_	sign?
*Initial phase*:	2009		2010	
Duration	1.444	n.s.	**2.6402**	*
Speed	**13.308**	***	**4.5179**	**
Steadiness	1.201	n.s.	0.9930	n.s.
*Homing phase*:	2009		2010	
Speed	**2.3976**	*	2.1965	n.s.
Steadiness	0.7679	n.s.	1.6598	n.s.
Efficiency	0.8857	n.s.	1.5421	n.s.
Correlation dimension	**2.8322**	*	1.5753	n.s.

**Table 4:** Significant differences between individual pigeons: by least squared means using the Tukey method for multiple comparisons.

Year	bird	median	bird	median	sign?	bird	median	bird	median	sign?
Duration (s) of intial phase										
2010	07–402	533	08–779	75	*	08–779	75	07–402	533	*
Flying speed (km/h) during the initial phase										
2009	06–235	60	06–214	50	***	06–249	49	06–235	60	***
			06–232	51	***			06–233	57	**
			06–249	49	***			06–213	56	**
			06–254	51	***			06–243	56	*
	06–233	57	06–214	50	***	06–214	50	06–235	60	***
			06–232	51	**			06–233	57	***
			06–249	49	**			06–213	56	***
			06–254	51	**			06–243	56	**
	06–213	56	06–214	50	***	06–232	51	06–235	60	***
			06–232	51	**			06–233	57	**
			06–249	49	**			06–213	56	**
			06–254	51	*			06–243	56	**
	06–243	56	06–214	50	**	06–254	51	06–235	60	***
			06–232	51	**			06–233	57	**
			06–249	49	*			06–213	56	*
			06–254	51	*			06–243	56	*
2010	08–755	60	07–393	45	**	07–393	45	08–755	60	**
			07–405	50	*			07–402	58	**
	07–402	58	07–393	45	*			08–797	56	**
	08–797	56	07–393	45	**	07–405	50	08–755	60	*
Correlation dimension during the homing phase										
2009	06–213	3.80	06–235	2.67	*	06–235	2.67	06–213	3.80	*
	06–254	3.62	06–235	2.67	*		2.67	06–254	3.62	*

Sign., significant differences

***, p < 0,001

**, p < 0.01

*, p < 0.05

n.s. not significant

Not surprisingly, the most pronounced differences concern the flying speed during the initial phase. The birds’ medians range from 49 to 60 km/h (see Table 1). Four of the 2009 birds initially fly significantly faster than the four others, and in 2010, pigeon 07–393, with only 45 km/h, is significantly slower than the three fastest birds. During the homing phase, all pigeons have significantly higher speeds (T = 0, both groups, p < 0.001, Wilcoxon test), ranging from 55 to 70 km/h. The differences between individual birds become smaller and, although the ART ANOVA indicates significant differences, the ones between bird 06–235 on the one side and 06–232 and 06–254 on the other side just do not reach significance (0.1 < p < 0.05).

The duration of the initial phase varies greatly, with the birds on average staying about 165 and 263 s before deciding to head home. Pigeon 07–402 needed exceedingly long to depart, with a median of about 9 min to reach the Point of Decision, while the fastest bird 08–779 took, on average, only 75 s before it started its homing flight. Lingering around after being released appears to be a personal trait of bird 07–402, but this bird is among those with the highest flying speed during the homing phase.

Steadiness increased markedly from medians of 0.22 and 0.19 during the initial phase to medians of 0.76 and 0.74 during the homing phase (T = 0, both groups, p < 0.01), but differences between individual birds are not indicated. There are also no significant differences in efficiency, with medians of 0.78 and 0.76—all birds appear to be similar in this aspect. This, however, may not be too surprising, considering the modest distances involved and given that all our pigeons are descendants of racing pigeons, with their ancestors having experienced a strong selection for fast and efficient homing over numerous generations.

The correlation dimensions cover a wide range, with medians of 3.30 and 3.37, respectively, in the two groups. Individual medians range from 2.67 up to 3.80; the values from single releases are as low as 2.31 and as high as 5.22 (see Table 2). The birds of the 2010 group do not differ significantly, but there are interesting differences among the 2009 birds (Table 3). With 2.67, pigeon 06–235 has the lowest correlation dimension, significantly lower than those of the two birds with the highest medians (see Table 4), with values at the different releases mostly below 3.0. At the same time, this pigeons has the highest flying speed during the initial phase as well as during the homing phase (see Tables 1 and 2). Pigeon 06–254, in contrast, has a median correlation dimension of 3.80; the values of its individual flights are all above 3.3, during one flight even above 4.0.

The correlation dimensions reflect the numbers of inputs in a system and thus its complexity (see [9] for details and Supporting Information S2 Text). Hence the observed differences suggest that not all pigeons use the same amount of information to navigate or interpret them differently–bird 06–254 appears to use more complex information in its navigational processes than e.g. 06–235. The correlation dimensions of 06–235 are so low that they could be interpreted as suggesting that this bird includes fewer factors in the navigational process.

The navigational ‘map’ is a multi-factorial system (see [11,16–19]). Recent studies indicate that pigeons can navigate successfully when one of the factors is not included: e.g., within a strong magnetic anomaly, birds were initially disoriented, but later became oriented while still within the anomaly—we interpreted this that they found the local magnetic information to be unreliable, disregarded it and concentrated on other, non-magnetic cues [20]. Also, when the magnetic field was disrupted by stronger temporal variations, the correlation dimension decreased significantly, suggesting that the birds disregarded the magnetic field in this situation, but this did not affect their efficiency [11]. Cutting the ophthalmic nerve that transmits magnetic information to the brain likewise did not disrupt orientation (e.g. [21]). How the low correlation dimension of 06–235 is to be interpreted and what using less complex information could mean is not entirely clear.

Altogether, we would not have expected large differences between pigeons, as they all mastered the homing task successfully. Nevertheless, our findings indicate some individual differences. The lack of significant differences in steadiness and efficiency shows that all individuals perform the homing flight equally well; it is also a great documentation of the general robustness of the pigeons’ navigational strategy, at least in the short distances involved in this study. The differences in the correlation dimension, however, appear to indicate individual differences in the navigational process, with some birds possibly utilizing more information than others or at least weigh the navigational factors differently. This is unexpected, since all birds have a very similar background concerning their early experience, at least as far as we could control it; it seems possible, however, that some pigeons made more extended spontaneous excursions than others when they were released for free flights at the loft. All birds should be equally familiar with the navigational factors in their home region by the training program, but some may interpret the regional factors differently. The observed differences in correlation dimension could reflect individual traits.

### Correlations between the variables

In a next step, we looked for correlations between the variables of either phase and between phases; coefficients of correlation are listed in Table 5.—Only in two cases, we find a correlation that is significant in both groups. One is a positive correlation between the flying speed of the two phases, suggesting that some birds generally tend to fly a bit faster than others, probably reflecting their athletic condition. The other is a strong correlation between steadiness during the homing phase and efficiency, a very close relationship that is to be expected, because both variables increase as the flight becomes straighter. The steadiness of the initial phase and that of the homing phase are not correlated, however.

**Table 5:** Correlations between variables: Spearman Rank coefficients of correlation.

Variables		2009		2010	
*Initial phase*:					
Duration	flying speed	**0.630**	*	0.338	n.s.
Duration	steadiness	- 0.214	n.s.	- 0.450	n.s.
Steadiness	flying speed	0.321	n.s.	0.538	n.s.
*Cross phase correlations*:					
Duration I	flying speed H	0.476	n.s.	0.050	n.s.
Duration I	steadiness H	- 0.214	n.s.	0.150	n.s.
Duration I	efficiency H	- 0.018	n.s.	0.067	n.s.
Duration I	correl. dim. H	0.214	n.s.	- 0.267	n.s.
Flying speed I	flying speed H	**0.690**	*	**0.808**	**
Flying speed I	steadiness H	0.048	n.s.	- 0.367	n.s.
Flying speed I	efficiency H	0.042	n.s.	0.383	n.s.
Flying speed I	correl. dim. H	0.119	n.s.	**- 0.921**	***
Steadiness I	flying speed H	- 0.310	n.s.	**0.625**	*
Steadiness I	steadiness H	0.411	n.s.	- 0.417	n.s.
Steadiness I	efficiency H	0.280	n.s.	- 0.358	n.s.
Steadiness I	correl. dim. H	- 0.008	n.s.	- 0.533	n.s.
*Homing phase*:					
Flying speed	steadiness	- 0.470	n.s.	- 0.075	n.s
Flying speed	efficiency	- 0.482	n.s.	- 0.025	n.s.
Flying speed	correl. dim.	- 0.238	n.s.	**- 0.725**	*
Steadiness	efficiency	**0.952**	***	**0.992**	***
Steadiness	correl. dim.	**0.827**	**	0.383	n.s.
Efficiency	correl. dim.	**0.923**	**	0.417	n.s.

Correl. dim. correlation dimension. I, data from the initial phase; H, data from the homing phase. Significant correlations are given in bold script, with the significance level indicated

***, p < 0.001

** p < 0.01

*, p < 0.05

n.s, not significant

In six cases, a correlation is significant for one group only. In case of steadiness during the initial phase and flying speed during the homing phase, the coefficients of correlation have different signs, suggesting that here the significance could have occurred by chance. This could also to be true for the flying speed during the initial phase and the correlation dimension, where the 2010 group shows a rather strong correlation between the two variables—here, the relationship between the two variables is entirely unclear.—In the other four cases, the coefficients of the significantly correlated samples and the non-significant samples have the same signs, suggesting there could be a weak correlation, which does not become significant in the one group. With due caution, we will interpret these relationships.

**Fig 3:**
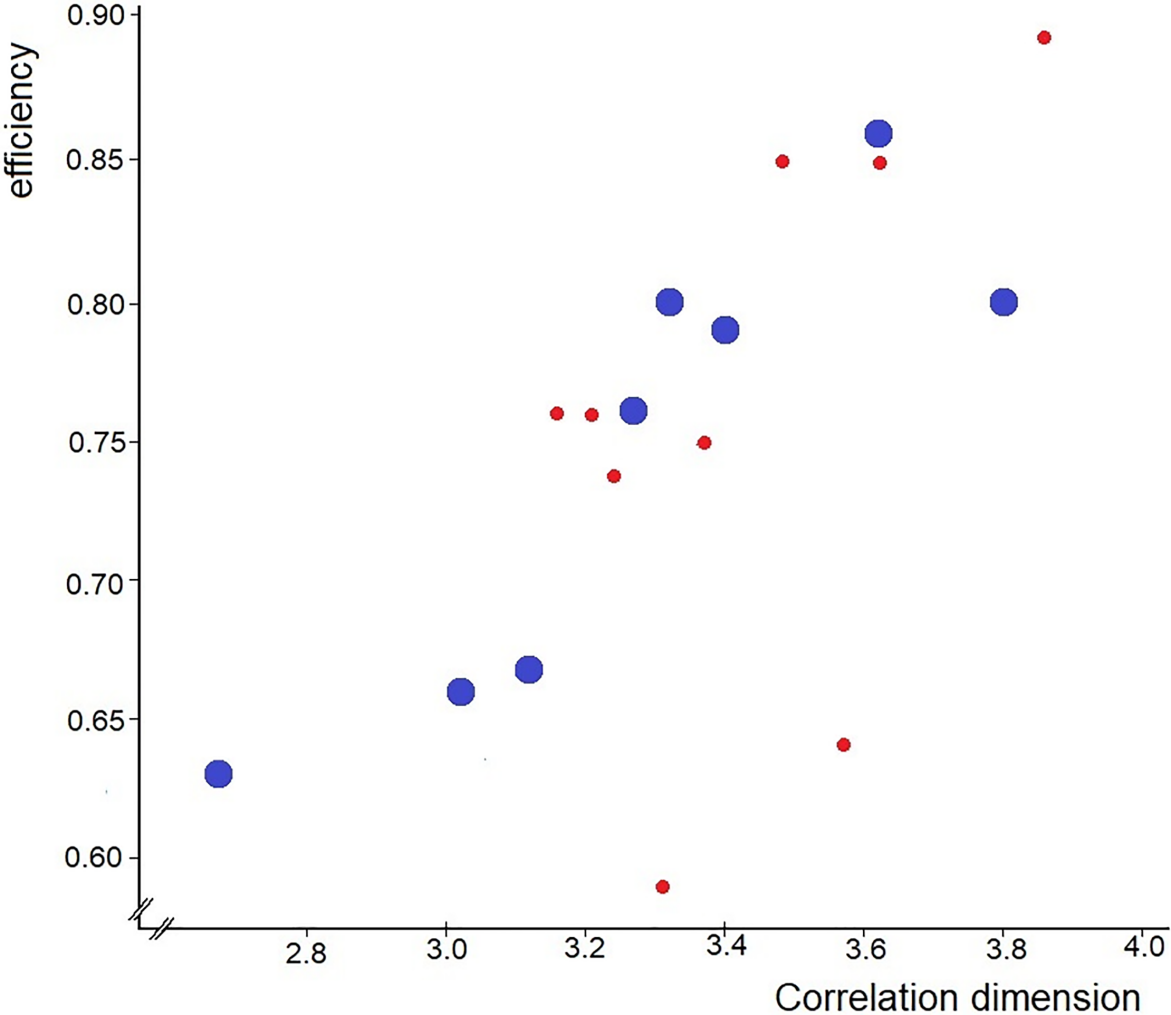
Positive relationship of the median correlation dimension of individual pigeons and their median efficiency. Larger blue symbols: 2009 birds, where the correlation is significant; smaller red symbols: 2010 birds.

During the initial phase, the duration seems positively correlated with the flying speed, which could mean that birds that fly faster at the release site stay there longer, possibly checking the local factors more intensively. The other three cases concern the homing phase. The flying speed seems negatively correlated with the correlation dimension, suggesting that pigeons using more complex navigational information tend to fly slightly slower. The others cases are positive correlations of steadiness and of efficiency with the correlation dimension, which are significant in the 2009 group, but do not reach significance the 2010 group (Fig 3). Such a correlation would indicate that pigeons using more complex navigational information fly more steadily and more efficiently. This could be a true correlation, since a similar relationship between the correlation dimension and steadiness was already suggested in an earlier study [9]. It may turn out to be a general feature of pigeon navigation: using more complex navigational information appears to pay off!

## General discussion

Tracks of homing pigeons have been recorded since the turn of the century (see [5]), but detailed in-depth analyses of the data obtained this way are still rare.

The Oxford group in England recorded a considerable number of tracks mainly in the vicinity of the loft; they analyzed them mostly in view of route stereotypies (e.g. [22–24]) and the influence of landscape structure, trying to identify landmarks used by pigeons (e.g. [25–27]), but also in view of the spatial entropy [28]. Their published data show many parallels to our findings: Flying speed and efficiency largely agree with ours, and although the authors do not explicitly distinguish between an initial phase and a homing phase, low efficiencies near the point of release indicate the existence of an initial phase in their tracks [29]. For birds homing repeatedly over 10 km, they obtained flying speeds of individual birds between 59.2 and 65 km/h and efficiencies between 0.82 and 0.90 [25]–the flying speeds are similar, the efficiency slightly larger than in the present study, which is most likely due to the fact that their birds were highly familiar with the respective site, having been released on 20 consecutive days from the same site.

The kind of analysis presented here, considering individual difference between birds and the correlation of variables on the basis of tracks, is a novel approach. Indications for individual traits in pigeons have already been obtained before, however, by direct observation with binoculars. Füller and Kowalski [30], releasing birds more than 50 times from the same sites, described significant differences in the vanishing directions, suggesting that the individual pigeons’ ‘ideas’ about the home direction and the optimal route to be taken differed slightly. When tracking the homing flights became possible, published tracks of birds homing repeatedly from the same sites documented that individual birds preferred somewhat different routes (e.g. [22–24]). When Frankfurt pigeons were repeatedly released from a site due west, some individuals preferred more northerly, others direct and even others more southerly routes [30]. In the present analysis, since each release site was used only once, we cannot assess whether the differences in the routes taken are chance or have a systematic reason. The observed differences between individuals in the correlation dimension are surprising, as they appear to suggest individual traits in the navigational processes.

We have to consider, however, that the data set for this analysis, because of the requirement that all bird fly the same routes on the same day, is limited. It is a first approach; using more birds and more releases might have given a more complete picture, with additional differences between individuals and weaker correlations could possibly have become significant. Another important point concerns the distances involved: Our study took place in the home region not far from the loft, well within our traditional training range, where the pigeons should be more or less familiar with the general course of the navigational factors. It is an interesting question to what extend our present findings can be generalized to longer distances and unfamiliar regions. Possibly, some relations could be modified if the pigeons have to interpret the navigational factors in a novel area where the specific values of these factors are very different from what they ever experienced before. And it would be intriguing to see what birds like 06–235 would do. They appear to get along with fewer factors in the familiar region and home just as efficiently as the other birds–would they get along with the same lower amount of navigational information, would they begin to use more complex information in such a situation, or would they get lost?–Hopefully, future studies will tell.

## Methods

The tracks analyzed here were recorded in spring and summer 2009 and 2010 on sunny days with little or no wind according to the laws and regulations for animal protection in Germany; the study was performed under the licenses V54 19v20/15 –F104/50 and V54 19v20/15 –F104/55 issued by Regierungspräsidium Darmstadt, Land Hessen (the responsible authority) and approved by their ethic committee.

### Test birds and test sites

The birds were bred and housed at the pigeon loft of the University of Frankfurt, where they were fed a commercial grain mixture and released for daily exercise flights except on testing days. The birds of this study were experienced pigeons two or three years old (see Tables 1 and 2). In their first year of life, they had taken part in our standard training program up to 40 km in the cardinal compass directions, and had homed singly in various test releases from different sites with or without carrying a flight recorder. Hence all release sites–between 9.3 and 23.5 km from the loft (Fig 1; see Supporting Information S1 Table for details)–lay well within the training range, but the pigeons were released at those specific sites for the first time.

### GPS-based flight recordings

We used GPS flight recorders, the same devices as in previous studies (see e.g. [13]), with the receiver modules being either Fasttrax up300 or up500 models, both allowing to acquire a fix of the current position with a precision of ±1.8 m. The mass of the complete recorder, including the Lithium polymer storage battery with a lifetime of up to 7 h, was 23 g. The recorder was set to take a positional fix every second.

The pigeons wore a harness made from Teflon tape (see [31] for details). The recorder was wrapped in anti-electrostatic plastic for shielding from external influences and also for shielding the pigeons from possible influences of the device. Access to satellite signals was secured by a window of the size of the patch antenna that was covered with normal plastic wrap. Immediately before release, the recorder was attached to the dorsal plate of the harness and fixed with Velcro and additional sticky tape. Harness and coating added another 7 g to the load.

The data analyzed include 84 tracks, all of them complete; they had also been included in the meta-analysis by [13]. In some cases, a pigeon joined another, and the birds flew together for major portions of the homing flight (see Supporting Information S1 and S2 Figs for details). This may cause some concern about non-independence, as the birds may have influenced each other. Since all birds had been released singly, no such effect is to be expected in the initial phase. An analysis of the data of the homing phase does not suggest a modification by flying together, and the rather high correlation dimensions also indicate that each bird was navigating on its own. Hence we decided not to exclude these data from the analysis.

### Data analysis and statistics

We divided the tracks by the 1st *Point of Decision* and determined the variables listed above for each phase of each individual flight. The calculation of the correlation dimension is given in more detail in Supporting Information S2 Text. For statistical analysis of the flight profiles of individual birds, we used Aligned Rank Transformed (ART) ANOVA using a linear mixed effects model, with the Bird Number as a fixed effect and the release site as a random effect [32]. This type ANOVA does not require the data to be normally distributed; we did, however, confirm that the variances of the samples were homogeneous. For post hoc comparison, we employed least squared means using the Tukey method for multiple comparisons. Further testing was based on the medians of the individual birds. We used the Spearman Rank Correlation to look for correlations between the variables, the Mann Whitney test to compare male and female pigeons and the Wilcoxon test to compare the flying speed and steadiness between phases.

## Supporting information

S1 Table Details on the releases. (PDF)

S2 Table Data of the individual flights. (PDF)

S1 Fig Tracks recorded in 2009. (PDF)

S2 Fig Tracks recorded in 2010. (PDF)

S1 Text Determining the points of decision. (PDF)

S2 Text Mathematical analysis. (PDF)
