# Correction: Behavioural traits of individual homing pigeons, *Columba livia* f. *domestica*, in their homing flights

**DOI:** 10.1371/journal.pone.0208170

**Published:** 2018-11-21

**Authors:** The PLOS ONE Staff

There are errors in both the Data Availability section and in Table 1. The publisher apologizes for the errors.

In the Data Availability section, the DOI for the Movebank Data Repository is listed incorrectly. The correct DOI is: 10.5441/001/1.pk8g6s2h.

In Table 1, the values in both rows labelled “Medians” are placed in incorrect columns. Please see the corrected Table 1 here.

**Table 1:** Initial phase: Medians and, in parentheses, range of the data for individual pigeons.

Pigeon	Sex	Duration (s)	Speed (km/h)	Steadiness
06–213	m	383 (45; 615)	56 (53; 59)	0.17 (0.07; 0.49)
06–214	m	143 (60; 405)	50 (43; 52)	0.22 (0.14; 0.46)
06–232	f	240 (45; 540)	51 (43; 52)	0.22 (0.09; 0.40)
06–233	f	180 (90; 390)	57 (51; 62)	0.22 (0.11; 0.42)
06–235	m	293 (120;1410)	60 (56; 61)	0.26 (0.10;0.36)
06–243	m	113 (60; 180)	56 (52; 60)	0.36 (0.27; 0.67)
06–249	m	90 (45; 255)	49 (48; 56)	0.18 (0.12; 0.61)
06–254	f	150 (45; 870)	51 (48; 53)	0.28 (0.10; 0.43)
Medians	-	165	54	0.22
Data of 2010, 4 flights per bird				
07–357	m	263 (180; 825)	53 (51; 54)	0.16 (0.08; 0.24)
07–387	f	218 (105; 345)	54 (47; 62)	0.24 (0.09; 0.38)
07–389	f	465 (210; 735)	55 (52; 61)	0.08 (0.07; 0.16)
07–393	f	368 (150; 405)	45 (43; 51)	013. (0.08; 0.48)
07–402	m	533 (240;1515)	58 (54; 61)	0.19 (0.06; 0.27)
07–405	m	210 (45; 405)	50 (45; 54)	0.18 (0.12; 0.65)
08–755	m	345 (75; 630)	60 (53; 64)	0.37 (0.04; 0.50)
08–779	m	75 (60; 150)	50 (48; 54)	0.22 (0.08; 0.24)
08–797	m	105 (45; 315)	56 (50; 60)	0.26 (0.12; 0.28)
Medians	-	263	54	0.19

The 1^st^ column gives the pigeon’s individual number, with the first two digits indicating the year when it was born. *m*, *f*: male and female pigeons. The following columns give the median and, in parentheses, the minimum and the maximum recorded. *Duration* indicates the duration of the initial phase in seconds from the moment of release to the first Point of Decision; *Speed* gives the average flying speed during the initial phase in km/h; *steadiness* indicates the steadiness of the flight, given as mean vector lengths resulting from the headings of the various 1s-parts between two fixes.
