# Supplementary material for: Behavioural traits of individual homing pigeons, Columba livia f. domestica, in their homing flights
Source: PLoS One. 2018 Sep 27;13(9):e0201291. doi: 10.1371/journal.pone.0201291 (PMC6160002; doi:10.1371/journal.pone.0201291)
Supplement: S1 Table — (PDF) [file pone.0201291.s001.pdf]

# Supporting Information

## Name and position of the release sites, date of flights, Ap-value

### Behavioral traits of individual homing pigeons, *Columba livia* f. *domestica*, in their homing flights

Ingo Schiffner, Patrick Fuhrmann, Juliana Reimann and Roswitha Wiltschko

**Table S1 Name and position of the release sites, date of flights, Ap-value**

| | Abr. | Site | Home dir. | Distance (km) | Date | Ap-Index |
|---|---|---|---|---|---|---|
| 2009 | WAL | Wallau | 67° | 21.5 | 29.07.2009 | 2 |
| | GH | Gräfenhausen | 14° | 21.6 | 31.07.2009 | 4 |
| | KST | Königstädten | 43° | 23.5 | 04.08.2009 | 3 |
| | MT | Maintal | 263° | 11.7 | 19.08.2009 | 8 |
| | HOF | Hofheim | 83° | 13.3 | 27.08.2009 | 6 |
| | SB | Steinbach (Ts.) | 134° | 9.3 | 31.08.2009 | 5 |
| 2010 | KB | Kelsterbach | 56° | 10.3 | 25.05.2010 | 3 |
| | RB | Rosbach | 194° | 19.2 | 14.06.2010 | 4 |
| | NA | Neu-Ansbach | 154° | 21.3 | 22.06.2010 | 4 |
| | BGR | Burggräfenrode | 214° | 17.3 | 24.06.2010 | 4 |

Home dir., home direction. Ap is a geomagnetic index, characterizing the fluctuation of the geomagnetic field during the respective day (see reference for details).

Schiffner I, Wiltschko R. (2011) Temporal fluctuations of the geomagnetic field affect pigeons' entire homing flight. J. Comp. Physiol. A, 197, 765-772.
