# Supplementary material for: Behavioural traits of individual homing pigeons, Columba livia f. domestica, in their homing flights
Source: PLoS One. 2018 Sep 27;13(9):e0201291. doi: 10.1371/journal.pone.0201291 (PMC6160002; doi:10.1371/journal.pone.0201291)
Supplement: S2 Table — (PDF) [file pone.0201291.s002.pdf]

Supporting Information

Data of the individual flights

Behavioral traits of individual homing pigeons, *Columba livia* f. *domestica*, in their homing flights

Ingo Schiffner, Patrick Fuhrmann, Juliana Reimann and Roswitha Wiltschko

Data of the individual flights

Duration of the Initial Phase (s)

**2009**	WAL	GH	KST	MT	HOF	SB	Median
06-213	150	615	45	555	600	210	383
06-214	405	60	105	135	210	150	143
06-232	240	45	540	120	240	465	240
06-233	225	390	255	135	135	90	180
06-235	1410	120	150	585	180	405	293
06-243	180	120	75	60	105	135	113
06-249	60	120	165	60	255	45	90
06-254	150	285	150	60	870	45	150
Median	203	120	150	128	225	143	

**2010**	KB	RB	NA	BGR	Median
07-357	825	255	270	180	263
07-387	345	105	195	240	218
07-389	570	210	735	360	465
07-393	405	150	360	375	368
07-402	1515	390	675	240	533
07-405	45	165	405	255	210
08-755	75	630	585	105	345
08-779	60	60	90	150	75
08-797	45	90	120	315	105
Median	345	165	360	240	

Fying speed during the Initial Phase (km/h)

**2009**	WAL	GH	KST	MT	HOF	SB	Median
06-213	59	56	53	58	56	54	56
06-214	52	46	43	51	50	49	50
06-232	51	43	52	48	51	52	51
06-233	62	54	60	56	58	51	57
06-235	58	56	60	60	59	61	60
06-243	60	55	52	56	56	55	56
06-249	48	49	56	48	55	48	49
06-254	52	51	51	48	53	49	51
Median	55	53	53	54	56	52	

**2010**	KB	RB	NA	BGR	Median
07-357	51	54	53	52	53
07-387	62	47	52	56	54
07-389	61	52	54	56	55
07-393	45	43	51	45	45
07-402	54	57	61	58	58
07-405	45	50	54	49	50
08-755	57	64	63	53	60
08-779	54	49	48	51	50
08-797	50	57	60	54	56
Median	54	52	54	53	

Steadiness during the Initial Phase

**2009**	WAL	GH	KST	MT	HOF2	SB	Median
06-213	0.09	0.13	0.20	0.07	0.26	0.49	0.17
06-214	0.18	0.25	0.14	0.16	0.39	0.46	0.22
06-232	0.25	0.15	0.09	0.40	0.25	0.18	0.22
06-233	0.16	0.21	0.39	0.22	0.11	0.42	0.22
06-235	0.10	0.18	0.32	0.19	0.36	0.34	0.26
06-243	0.38	0.29	0.59	0.67	0.34	0.27	0.36
06-249	0.13	0.13	0.41	0.23	0.12	0.61	0.18
06-254	0.43	0.24	0.10	0.25	0.38	0.30	0.28
Median	0.17	0.20	0.26	0.23	0.30	0.38	

Steadiness during the Initial Phase (continued)

**2010**	KB	RB	NA	BGR	Median
07-357	0.14	0.24	0.08	0.18	0.16
07-387	0.20	0.38	0.27	0.09	0.24
07-389	0.07	0.08	0.16	0.08	0.08
07-393	0.16	0.48	0.08	0.09	0.13
07-402	0.14	0.24	0.27	0.06	0.19
07-405	0.65	0.12	0.13	0.22	0.18
08-755	0.28	0.50	0.04	0.45	0.37
08-779	0.08	0.22	0.24	0.22	0.22
08-797	0.24	0.28	0.28	0.12	0.26
Median	0.16	0.24	0.16	0.12	

Fying speed during the Homing Phase (km/h)

**2009**	WAL	GH	KST	MT	HOF	SB	Median
06-213	57	57	60	68	63	55	59
06-214	48	51	60	69	59	55	57
06-232	52	53	56	64	57	60	57
06-233	59	55	62	64	57	62	61
06-235	60	54	65	65	64	62	63
06-243	52	54	62	70	58	56	57
06-249	55	54	57	59	63	62	58
06-254	53	54	55	61	64	55	55
Median	55	54	60	65	61	58	

**2010**	KB	RB	NA	BGR	Median
07-357	64	64	62	63	64
07-387	65	63	65	70	65
07-389	67	66	69	62	67
07-393	70	59	55	68	64
07-402	67	67	68	68	68
07-405	62	61	69	64	63
08-755	65	66	72	72	69
08-779	69	64	67	67	67
08-797	68	69	71	71	70
Median	67	64	68	68	

Steadiness during the Homing Phase

**2009**	WAL	GH	KST	MT	HOF	SB	Median
06-213	0.63	0.85	0.51	0.84	0.70	0.93	0.77
06-214	0.64	0.78	0.71	0.88	0.70	0.86	0.75
06-232	0.71	0.67	0.67	0.85	0.40	0.88	0.69
06-233	0.53	0.88	0.90	0.80	0.44	0.77	0.79
06-235	0.73	0.52	0.60	0.61	0.48	0.90	0.61
06-243	0.55	0.81	0.84	0.90	0.36	0.80	0.81
06-249	0.69	0.71	0.68	0.48	0.62	0.94	0.69
06-254	0.86	0.86	0.69	0.65	0.87	0.88	0.86
Median	0.67	0.80	0.69	0.82	0.55	0.88	

**2010**	KB	RB	NA	BGR	Median
07-357	0.84	0.84	0.46	0.86	0.84
07-387	0.39	0.60	0.55	0.72	0.58
07-389	0.62	0.75	0.90	0.71	0.73
07-393	0.87	0.94	0.50	0.88	0.875
07-402	0.70	0.74	0.76	0.79	0.75
07-405	0.84	0.64	0.49	0.55	0.60
08-755	0.77	0.67	0.61	0.85	0.72
08-779	0.89	0.80	0.74	0.85	0.825
08-797	0.69	0.91	0.61	0.78	0.74
Median	0.77	0.75	0.61	0.79	

Efficiency during the Homing Phase

**2009**	WAL	GH	KST1	MT	HOF	SB	Median
06-213	0.57	0.85	0.47	0.86	0.75	0.92	0.80
06-214	0.60	0.77	0.73	0.89	0.74	0.86	0.76
06-232	0.66	0.64	0.68	0.86	0.46	0.88	0.67
06-233	0.49	0.88	0.90	0.80	0.46	0.77	0.79
06-235	0.72	0.47	0.60	0.65	0.52	0.90	0.63
06-243	0.51	0.81	0.85	0.90	0.41	0.79	0.80
06-249	0.65	0.67	0.68	0.52	0.65	0.94	0.66
06-254	0.86	0.86	0.70	0.69	0.88	0.88	0.86
Median	0.63	0.79	0.69	0.83	0.59	0.88	

Efficiency during the Homing Phase (continued)

**2010**	KB	RB	NA	BGR	Median
07-357	0.85	0.85	0.48	0.86	0.85
07-387	0.40	0.62	0.56	0.74	0.59
07-389	0.63	0.77	0.91	0.73	0.75
07-393	0.89	0.94	0.52	0.89	0.89
07-402	0.72	0.76	0.76	0.80	0.76
07-405	0.85	0.66	0.56	0.61	0.64
08-755	0.78	0.69	0.61	0.86	0.74
08-779	0.90	0.84	0.74	0.86	0.85
08-797	0.72	0.92	0.61	0.79	0.76
Median	0.78	0.77	0.61	0.80	

Correlation dimension

**2009**	WAL	GH	KST	MT	HOF	SB	Median
06-213	4.30	2.78	3.76	3.28	3.91	3.84	3.80
06-214	3.70	3.27	5.03	3.03	3.27	2.93	3.27
06-232	4.92	3.53	2.88	3.32	2.93	2.68	3.13
06-233	2.47	4.03	3.53	3.44	2.97	3.36	3.40
06-235	3.09	2.63	3.32	2.71	2.44	2.56	2.67
06-243	3.32	3.10	3.71	3.34	3.10	3.32	3.32
06-249	3.24	3.11	3.60	2.78	2.60	2.92	3.02
06-254	4.05	3.32	3.31	3.73	3.50	3.96	3.62
Median	3.51	3.19	3.57	3.30	3.04	3.13	

**2010**	KB	RB	NA	BGR	Median
07-357	2.31	3.60	3.54	3.41	3.48
07-387	3.29	3.56	3.32	2.93	3.31
07-389	2.78	3.51	3.86	3.22	3.37
07-393	3.18	5.22	3.89	3.82	3.86
07-402	2.83	3.37	4.18	2.94	3.16
07-405	2.77	3.39	3.75	3.75	3.57
08-755	3.44	3.16	3.33	3.10	3.25
08-779	4.25	3.70	3.50	3.54	3.62
08-797	3.30	3.47	3.11	2.87	3.21
Median	3.15	3.54	3.57	3.25	
