# Supplementary material for: Behavioural traits of individual homing pigeons, Columba livia f. domestica, in their homing flights
Source: PLoS One. 2018 Sep 27;13(9):e0201291. doi: 10.1371/journal.pone.0201291 (PMC6160002; doi:10.1371/journal.pone.0201291)

# Supporting Information

## Tracks of individual pigeons recorded in 2009

### Behavioral traits of individual homing pigeons, *Columba livia* f. *domestica*, in their homing flights

Ingo Schiffner, Patrick Fuhrmann, Juliana Reimann and Roswitha Wiltschko

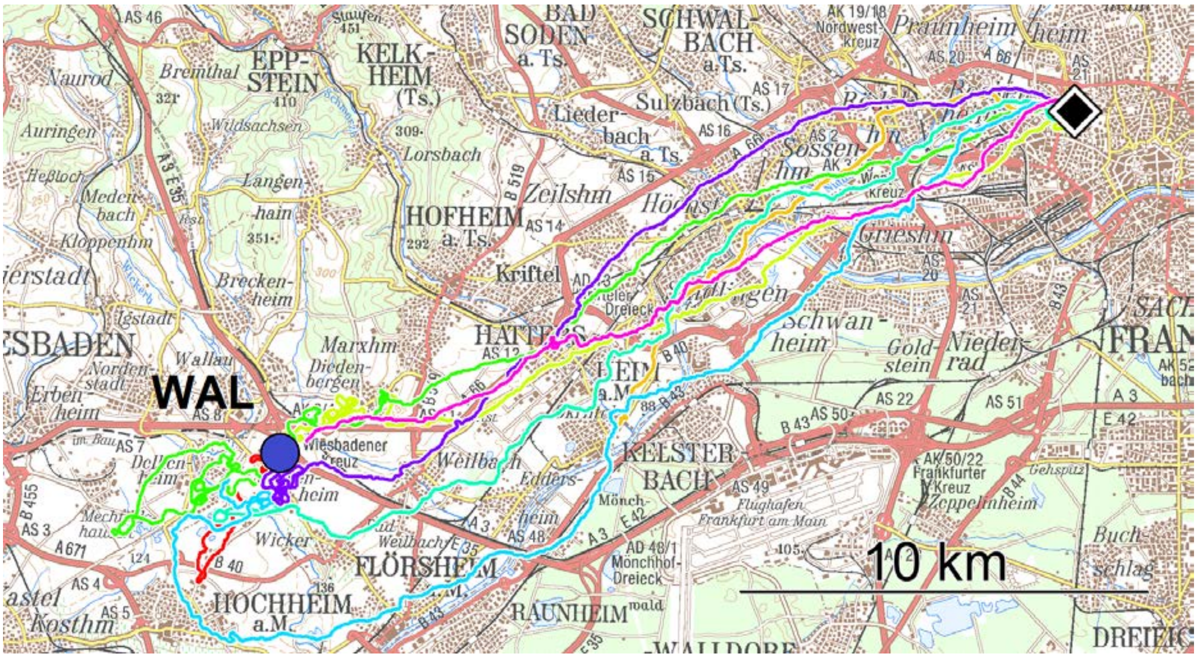


**Fig. S1. Tracks recorded in 2009.** Blue dots, release sites; black diamond, loft at Frankfurt am Main. The tracks of individual birds are given in different color, see legend. - From WAL, 06-249 and 06-213 as well as 06-243 and 06-214 flew together.

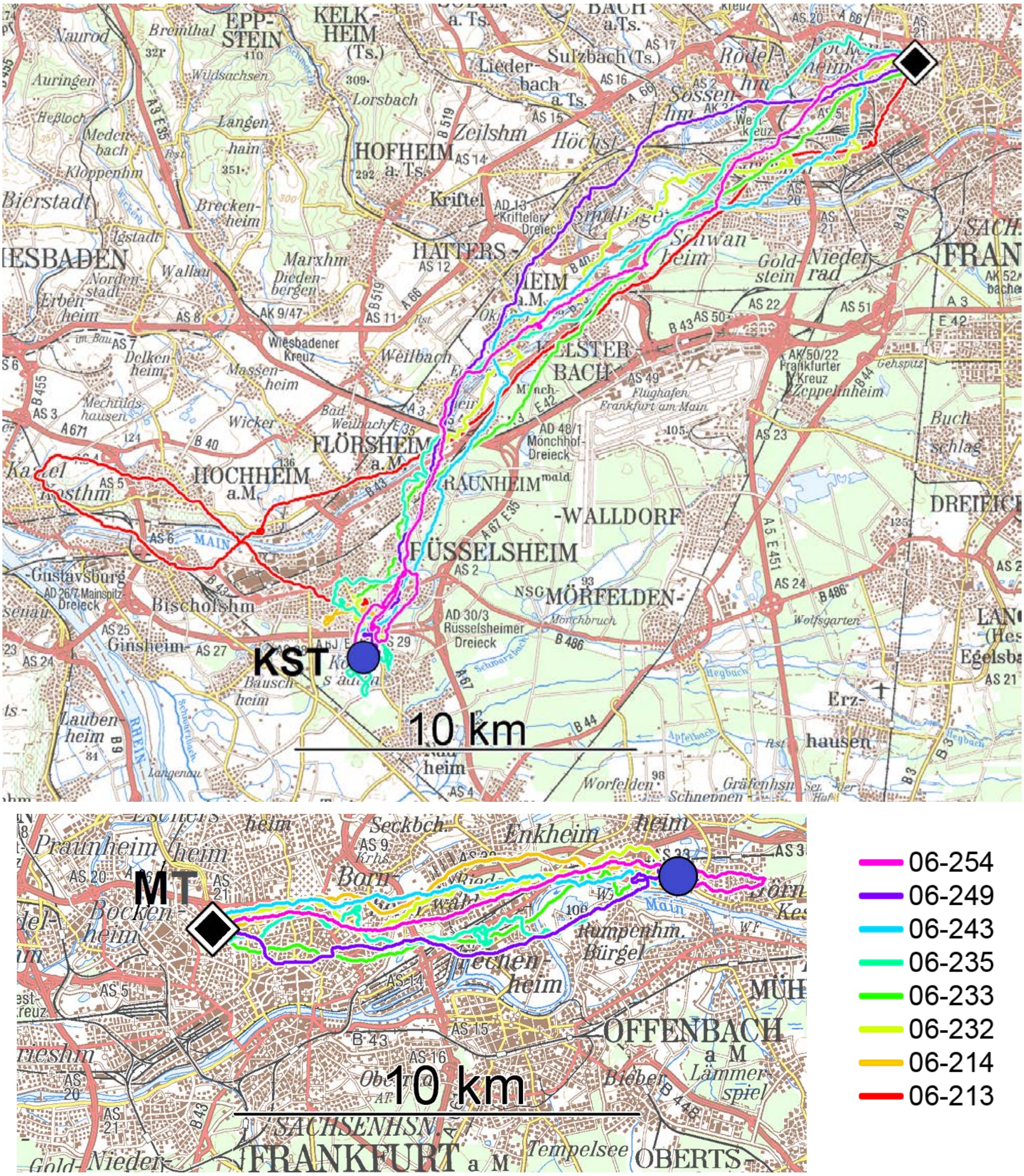


**Fig. S1. Tracks recorded in 2009.** Blue dots, release sites; black diamond, loft at Frankfurt am Main. The tracks of individual birds are given in different color, see legend. - From KST, 06-233 and o6-214 flew together, and from MT, 06-214 and 06-213 flew together. From GH,06-254 and 06-249 flew together; from HOF, 06.232 and 06-235 flew together and from SB, all birds flew singly.

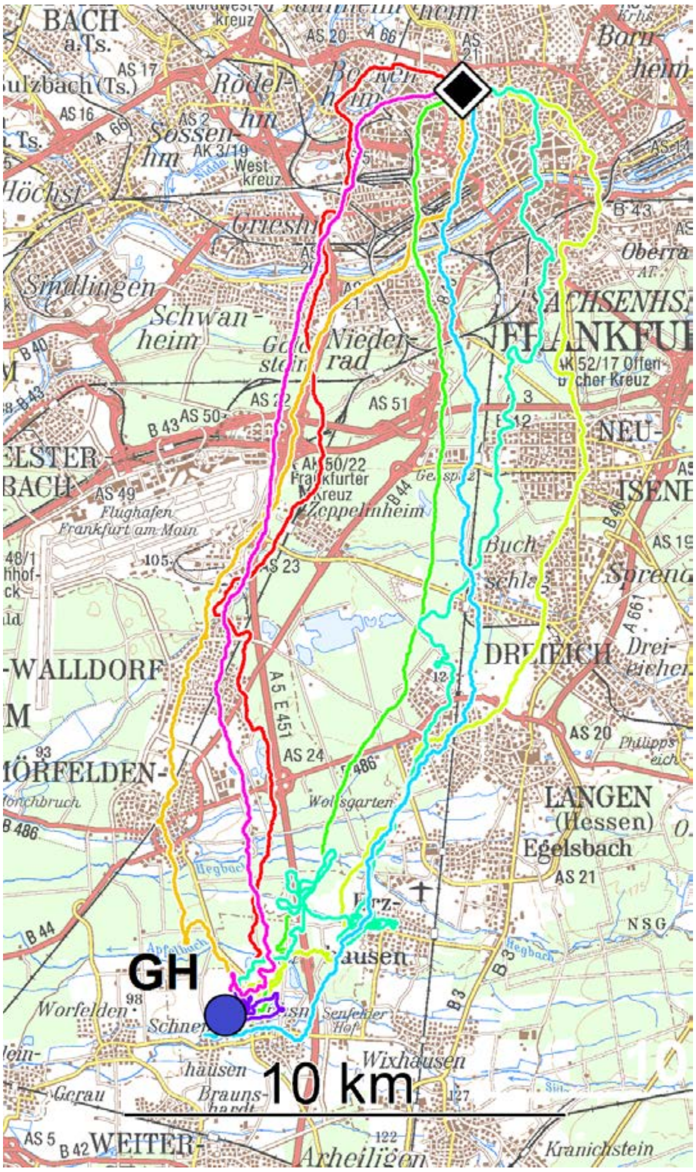


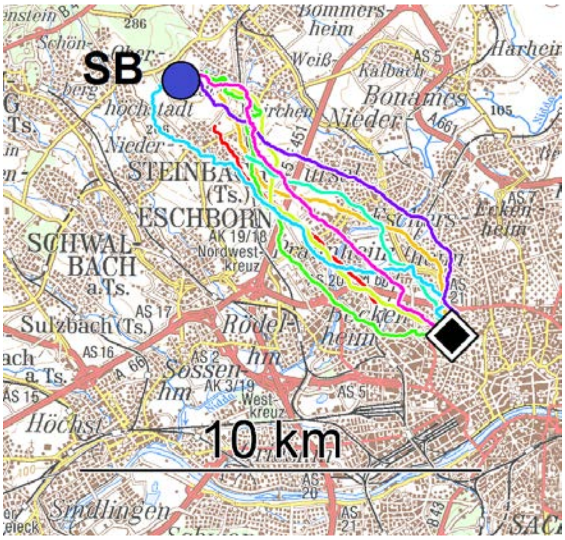


**Fig. S1. Tracks recorded in 2009**. Blue dots, release sites; black diamond, loft at Frankfurt am Main. The tracks of individual birds are given in different color, see legend. -. From GH,06-254 and 06-249 flew together; from HOF, 06.232 and 06-235 flew together and from SB, all birds flew singly.

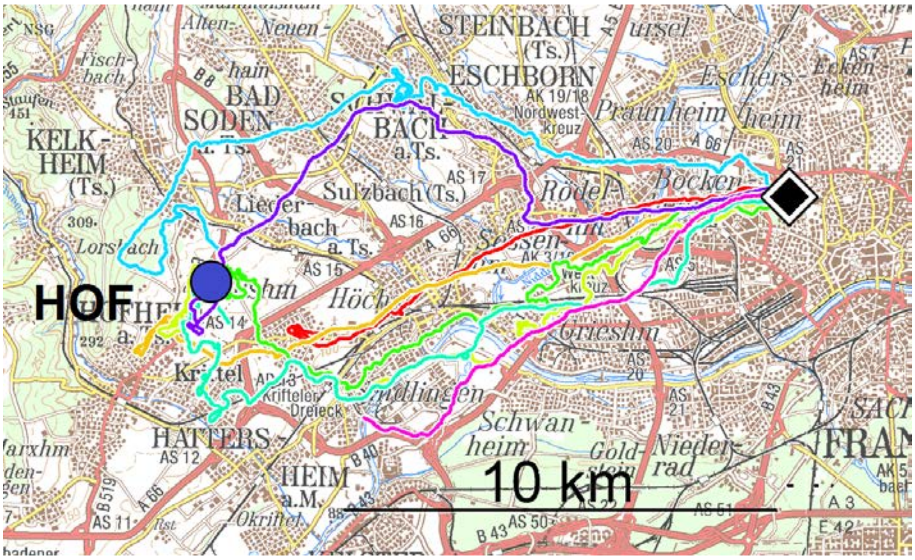


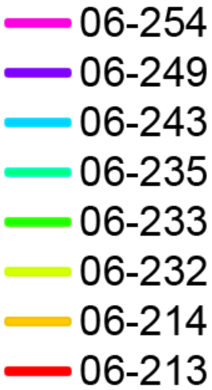

Supplement: S1 Fig — (PDF) [file pone.0201291.s003.pdf]
