# Supplementary material for: Behavioural traits of individual homing pigeons, Columba livia f. domestica, in their homing flights
Source: PLoS One. 2018 Sep 27;13(9):e0201291. doi: 10.1371/journal.pone.0201291 (PMC6160002; doi:10.1371/journal.pone.0201291)
Supplement: S2 Fig — (PDF) [file pone.0201291.s004.pdf]

Supporting Information

Tracks of individual pigeons recorded in 2010

Behavioral traits of individual homing pigeons, *Columba livia* f. *domestica*, in their homing flights

Ingo Schiffner, Patrick Fuhrmann, Juliana Reimann and Roswitha Wiltschko

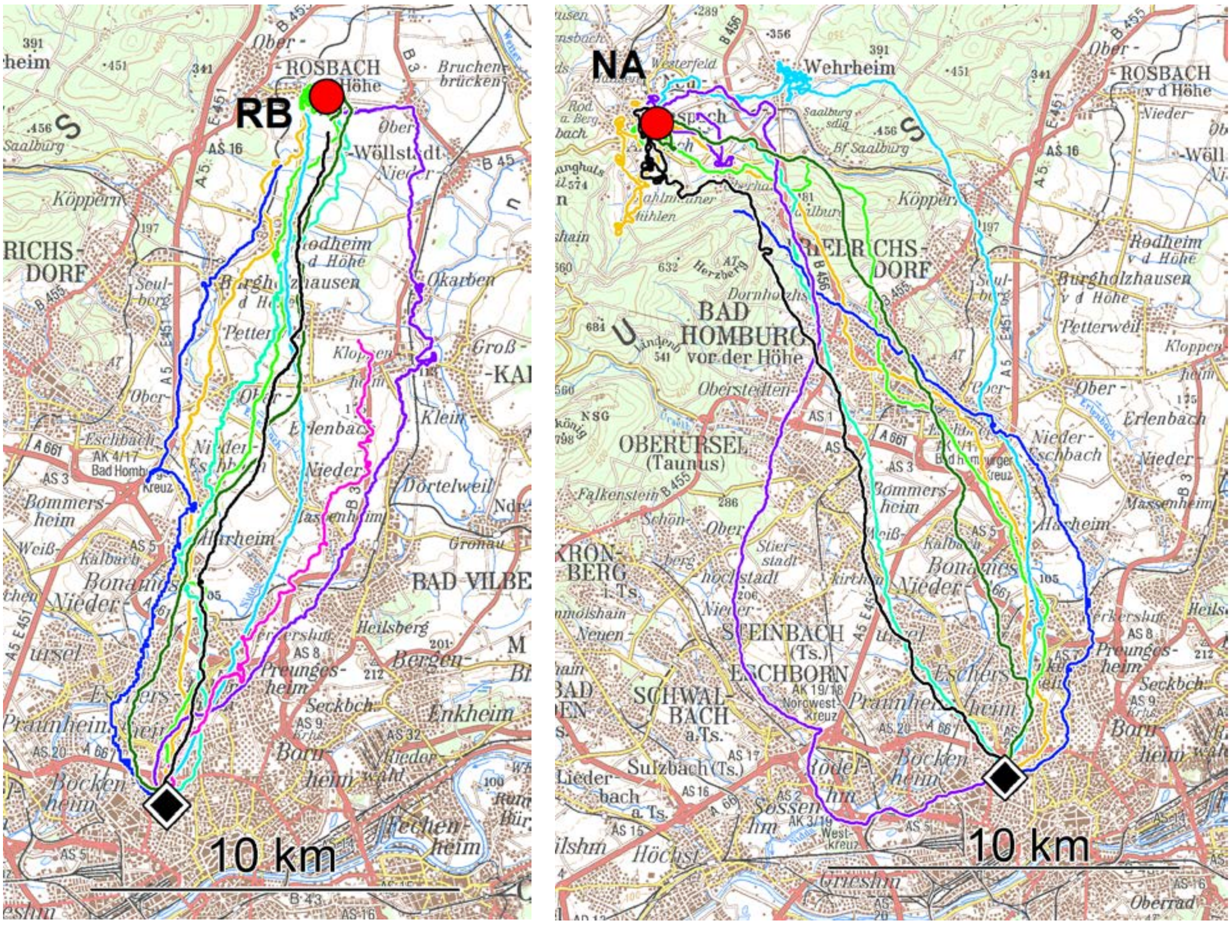

Fig.S2. Tracks recorded in 2010 (see also Fig. 2 in the main text). Red dots, release sites; black diamond, loft at Frankfurt am Main. The tracks of individual birds are given in different color, see legend.- From RB, 19.2 km from the loft, all birds flew singly; from NA, 21.3 km from the loft, birds 08-797 and 08-755 flew together.

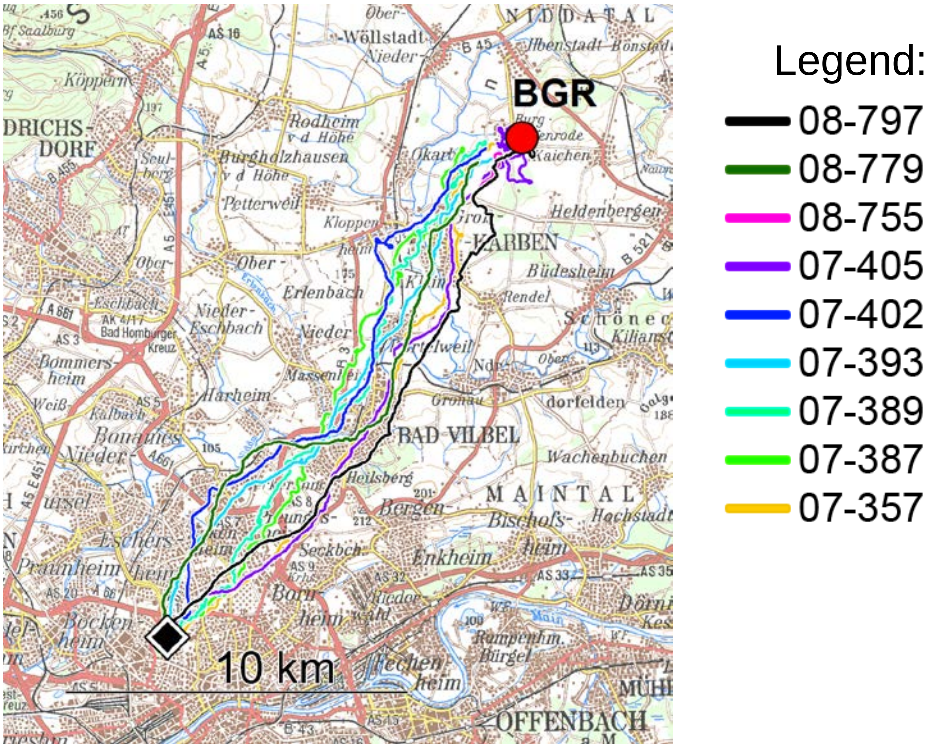

Fig.S2. Tracks recorded in 2010. Red dots, release sites; black diamond, loft at Frankfurt am Main. The tracks of individual birds are given in different color, see legend.- From BGR, 17.3. km, 08-755 and 08-797 flew together.
