# Supplementary material for: Behavioural traits of individual homing pigeons, Columba livia f. domestica, in their homing flights
Source: PLoS One. 2018 Sep 27;13(9):e0201291. doi: 10.1371/journal.pone.0201291 (PMC6160002; doi:10.1371/journal.pone.0201291)
Supplement: S1 Text — (PDF) [file pone.0201291.s005.pdf]

Supporting Information

Determining the Points of Decision

Behavioral traits of individual homing pigeons, *Columba livia* f. *domestica*, in their homing flights

Ingo Schiffner, Patrick Fuhrmann, Juliana Reimann and Roswitha Wiltschko

Determining the Points of Decision

For analysis, we divided the tracks in two phases, separated by a *Point of Decision* (see [1]:

Points of Decision are identified with the help of the following two variables:
(1) *cumulative velocity*, defined as the current distance from the release point divided by the time that had passed since release, and
(2) *steadiness*, here defined as the vector length of headings over a period of 60 s, calculated as a sliding mean every 15 s,
and *their changes over time.*

The highest increase in steadiness immediately before or during a period of increasing cumulative velocity marks a Point of Decision. The first one, usually close to the release point, indicates the moment when a pigeon decides to leave and head home (see Schiffner and Wiltschko 2009). When released at greater distances, the tracks often include more periods of increasing and decreasing cumulative velocity and steadiness so that additional Points of Decision can be determined in the same way, namely as the momentary highest increase in steadiness immediately before or during a period of increasing cumulative velocity that follows a period of decreasing cumulative velocity (for details, see [2].

The Points of Decision thus divide the tracks into several phases: the first one marks the end of the *initial* phase, where the pigeon flies around the release point, and the beginning of the *departure* phase where it leaves the site; the last one, if there are more than one, marks the beginning of the final *homing* phase.

References

1. Schiffner I, Wiltschko R (2009) Point of Decision: When do pigeons decide to head home? Naturwissenschaften 96:251-258.
2. Schiffner I, Baumeister J, Wiltschko R. (2011) Mathematical analysis of the navigational process in homing pigeons. J Theoret Biol 29:42-46.
