# Supplementary material for: Behavioural traits of individual homing pigeons, Columba livia f. domestica, in their homing flights
Source: PLoS One. 2018 Sep 27;13(9):e0201291. doi: 10.1371/journal.pone.0201291 (PMC6160002; doi:10.1371/journal.pone.0201291)
Supplement: S2 Text — (PDF) [file pone.0201291.s006.pdf]

Supporting Information

Mathematical analysis

Behavioral traits of individual homing pigeons, *Columba livia* f. *domestica*, in their homing flights

Ingo Schiffner, Patrick Fuhrmann, Juliana Reimann and Roswitha Wiltschko

Mathematical analysis: Implementation of algorithms

Dynamic systems theory is an area of mathematics focused on understanding and describing complex dynamical systems. In general, dynamic systems theory discerns three basic types of systems: deterministic, random and chaotic-deterministic systems. By observation of past states - provided a sufficient amount of observations are available - one can fully predict the behavior of a deterministic system. E.g., observing an ideal pendulum over an entire period provides sufficient knowledge about the pendulum's past states to fully predict any future states of the pendulum - it is a deterministic system. In contrast, when observing a dice, a random system, no amount of observations would grant the observer sufficient information about the behavior of this system – here, knowledge of past states of the system would never yield better predictions about future states than an educated guess. Chaotic-deterministic systems, although often confused with random systems, are essentially deterministic systems, as suggested by their name. Similar to deterministic systems, observation of past states allows an observer to make predictions about future states of the system. However, due to omnipresent uncertainty combined with a high sensitivity to initial states, which is typical for chaotic deterministic systems, prediction deteriorates over time until any prediction based on past states is as good as an educated guess, i.e. the system appears to behave randomly. A very common example for chaotic-deterministic systems is the weather: while weather forecasts tend to be reasonably accurate over short periods of time, long term predictions tend to deteriorate quickly.

Here we utilize the so-called method of time-lag embedding, a well-established method in dynamic system theory commonly used to characterize mechanical and mathematical systems (e.g. [1, 2]). According to Takens' embedding theorem [3], a dynamic system can be fully reconstructed in phase space given a series of observations of the state of the dynamic system, i.e. a time-series. In phase space, every degree of freedom or parameter of the system is represented as an axis in a multi-dimensional space. The number of degrees of freedom then is the minimum number of independent variables necessary to fully describe the system. The advantage of the method compared to other modelling approaches is that it requires no a priori knowledge of the underlying system like the number of factors involved and their specific interactions; instead the methods allow for the creation of a physical model of the process using only the recorded data. The method has been rigorously tested in mathematical systems where the exact number of degrees of freedom is known [4]; there, the number of degrees of freedom is equivalent to the number of terms in the equation. In physical/mechanical systems, it is the number of inputs, e.g. in the case of a robot navigating in a given environment, it is the number of independent sensors necessary to perform this task [2]. The same relationship applies to the pigeon's navigational process, with the degrees of freedom, as represented by the *correlation dimension*, indicating the number of

independent sensory modalities involved in the navigational process. Applied to tracks of pigeons the correlation dimension thus allows us to draw conclusions about the navigational strategy used [5].

The type of information used by the pigeons, i.e. route information, point-like information or geophysical gradients, and how this information is used, i.e. landmarks for piloting [6] or as elements of a mosaic map [7,8], has direct consequences on the degrees of freedom of the underlying navigational process, reflected by the correlation dimension. If pigeons e.g. use landmarks for piloting, then the underlying process is rather simple: the information provided is merely the path towards the next landmark—this type of information offers only one degree of freedom. Navigation based on a mosaic map, in comparison, requires the involvement of at least one additional factor, a compass. Hence it can be assumed that navigation based on a mosaic map would result in a higher dimensional process with a correlation dimension of at least 2. As the mosaic map is assumed to consist of static cues, each cue within the mosaic map is equivalent to every other, basically providing the same information. Multiple cues of the same type thus may increase certainty and allow pigeons to uniquely identify a specific cue, but essentially do not provide more information than a single cue alone.

If pigeons use multiple environmental gradients simultaneously as navigational cues, the degrees of freedom of the navigational process depend on the number of gradients involved, leading to a corresponding increase in the correlation dimension. Two different factors, one providing an equivalent to longitudinal and one providing an equivalent to latitudinal information, as well as one compass cue would suffice to allow pigeons to find their way home, hence the correlation dimension should be at least 3. The correlation dimension thus indicates the type of strategy applied: low correlation dimensions suggest simpler forms of navigation, like navigation based on point-like information, while higher correlation dimensions suggest navigation based on multiple environmental gradients. A marked change in the correlation dimension would then indicate a change in the navigational strategy and difference in correlation dimension between untreated control birds and experimental pigeons deprived of certain sensory information would indicate involvement of specific cues in the navigational process.

Mathematical analysis: Estimation of the Correlation Dimension

The correlation dimension is estimated by calculating the correlation integral $C^m(r)$:

$$C^m(r) = \frac{1}{M(M-1)} \sum_{i,j=1}^{M} \Theta(r - \|x_i - x_j\|)$$

with $i \neq j$, r the distance interval, m the embedding dimension

$$M = N - (m-1)\tau$$

with τ the embedding lag, m the embedding dimension and N the number of data points in the time series, where Θ denotes the so-called Heaviside step function:

$$\Theta(x) = \begin{Bmatrix} 1 & x \geq 0 \\ 0 & x < 0 \end{Bmatrix}$$

and $\|...\|$ denotes the so-called Euclidean norm, a measurement of distance in Euclidean vector space.

From the correlation integral the correlation dimension d_2 can then be obtained as the slope of

$\ln C^m$ versus $\ln r$:

with lnCm(r) $d_2 = \lim_{r \to 0} \lim_{m \to \infty} \frac{\ln C^m(r)}{\ln r}$

The slope of the curve is thus a direct measurement of the independent degrees of freedom or dimension of the system. In case of a navigational process these are the number of independent navigational factors used for navigation.

References

1. Small M (2005) Applied nonlinear time series analysis: applications in physics, physiology and finance (World Scientific Pub Co Inc.).
2. Nehmzow U (2006) Scientific methods in mobile robotics: quantitative analysis of agent behaviour' (Springer)
3. Takens F (1981) Detecting strange attractors in turbulence. In: Rand D, Young LS (eds) Dynamical Systems and Turbulence. Lecture Notes in Mathematics. 898. Springer, Berlin, pp 366-381.
4. Grassberger P, Procaccia I. (1983) Characterization of strange attractors. Physic Rev Lett 50:346-349
5. Schiffner I, Baumeister J, Wiltschko R. (2011) Mathematical analysis of the navigational process in homing pigeons. J Theoret Biol 29:42-46.
6. Griffin D R (1944) The sensory basis of bird navigation. Quartl Rev Biol 19:15-31.
7 Graue LC (1963) The effect of phase shifts in the day-night cycle on pigeon homing at distances of less than one mile. Ohio J Sci 63:214-217.
8. Wallraff HG (1974) Das Navigationssystem der Vögel. Ein theoretischer Beitrag zur Analyse ungeklärter Orientierungsleistungen. Oldenbourg, München, Wien.
